# Characterization of the Competitive Pneumocin Peptides of *Streptococcus pneumoniae*

**DOI:** 10.3389/fcimb.2019.00055

**Published:** 2019-03-12

**Authors:** Wei-Yun Wholey, Maha Abu-Khdeir, Emily A. Yu, Saher Siddiqui, Ogenna Esimai, Suzanne Dawid

**Affiliations:** ^1^Department of Pediatrics and Communicable Diseases, University of Michigan Medical School, Ann Arbor, MI, United States; ^2^Department of Microbiology and Immunology, University of Michigan Medical School, Ann Arbor, MI, United States; ^3^Department of Computer Science and Engineering, University of Texas at Arlington, Arlington, TX, United States

**Keywords:** *Streptococcus pneumoniae*, bacteriocin, colonization, competition, quorum sensing

## Abstract

In the polymicrobial environment of the human nasopharynx, *Streptococcus pneumoniae* (pneumococcus) competes with other members of the microbial community for limited nutrients in part by secreting small peptide bacteriocins called pneumocins. Pneumocin production is controlled by a quorum sensing system encoded by the *blp* locus. Although the locus is found in all pneumococci, there is significant variability in the repertoire of pneumocins and associated immunity proteins encoded in the Bacteriocin Immunity Region (BIR) and in the presence or absence of a functional Blp transporter. Strains without an active Blp transporter are inactive in plate overlay assays and rely on a homologous transporter that is only produced during brief periods of competence to stimulate the *blp* locus and secrete pneumocins. The variability of the locus suggests that selective pressure is influencing the content to promote the optimal competitive environment. Much of the variability in the *blp* locus has been described at the genome level; the phenotypic activity attributable to the various BIR genes has not been fully described. To examine the role of the predicted pneumocin peptides in competition, 454 isolates were screened for competence independent *blp* pheromone secretion using plate assays. Active strains were characterized for inhibition, BIR content, BlpC pherotype and serotype. Deletion analysis on inhibitory strains demonstrated that BlpI and BlpJ peptides function as a two-peptide bacteriocin and that BlpIJ immunity is encoded by the co-transcribed *blpU4/5* genes. BlpIJ secretion promotes inhibitory activity against the majority of pneumococcal isolates when expressed in a Blp transporter intact background. Intermediate levels of competition in biofilms were noted when BlpIJ containing strains carried the non-functional Blp transporter. Based on genome data, the combination of BlpIJ in a Blp transporter intact strain is surprisingly rare, despite clear advantages during colonization and biofilm growth. In contrast, we show that the *blpK/pncF* operon encoding the single-peptide pneumocin BlpK and its immunity protein is found in the majority of isolates. Unlike, BlpIJ and BlpK were shown to promote a limited spectrum of inhibition due in part to immunity that is independent of activation of the *blp* locus.

## Introduction

*Streptococcus pneumoniae* (pneumococcus) is a member of the human nasopharyngeal microbial community that can cause a wide spectrum of local and disseminated disease when mucosal barriers are breached. Because colonization precedes infection, survival in the nasopharynx is a crucial step in the pathogenesis of pneumococcal disease. The human nasopharyngeal microbial community is composed of a dynamic array of different bacterial species (Teo et al., 2015; Bosch et al., 2017; Kelly et al., 2017). Pneumococcal colonization is established during infancy and peaks in daycare age children (Teo et al., 2015; Bosch et al., 2017; Kelly et al., 2017). To survive in this polymicrobial environment and to compete for limited nutrients provided by the host, pneumococcus must actively compete with other members of the microbiome. One means of competition is through the secretion of bacteriocins, small antimicrobial peptides that typically target other closely related bacteria by disrupting either cell wall formation or membrane integrity. Bacteriocin secretion can eliminate competitors, freeing up valuable nutrients and space. Bacteriocin production is typically tightly regulated presumably to ensure that the peptides are only made under advantageous conditions (Nes et al., 1996; Wescombe et al., 2006; Cornforth and Foster, 2013; Maricic et al., 2016; Wholey et al., 2016; Shanker and Federle, 2017).

Pneumococci have been shown to encode a wide variety of bacteriocins, however, only a few loci have been shown to be inhibitory (Bogaardt et al., 2015). Inhibitory bacteriocins include the *blp* encoded pneumocins, the CibAB bacteriocin pair, and the pneumolancidins (Guiral et al., 2005; Dawid et al., 2007; Bogaardt et al., 2015; Maricic et al., 2016). The pneumocins are encoded by the *blp* locus, which is present in all sequenced pneumococcal isolates (Bogaardt et al., 2015; Miller et al., 2016). The *blp* locus contains genes that encode a peptide pheromone (BlpC), a two-component regulatory system (BlpRH), a dedicated transporter (BlpAB), a variable array of bacteriocin and immunity proteins within the bacteriocin immunity region (BIR), and some conserved accessory proteins whose functions are unknown but may play a role in immunity (de Saizieu et al., 2000; Dawid et al., 2007; Lux et al., 2007). The operons of the *blp* locus are upregulated in response to accumulation of the secreted extracellular BlpC pheromone, which activates the two-component system upon binding to the BlpH receptor. BlpC bound BlpH stimulates a phosphor-transfer reaction that results in an activated BlpR regulator. Activated BlpR binds to promoters in the locus and upregulates the operons in the BIR and the *blpABC* operon resulting in pneumocin secretion and positive feedback activation of the locus via increased pheromone secretion.

Although the *blp* locus itself is found in all pneumococcal genomes, there is significant variability in the content of the locus. There are four major allelic variants of the gene encoding the BlpC pheromone, designated BlpC_164_, BlpC_R6_, BlpC_6A_, and BlpC_T4_ in addition to minor variants found in a small number of strains (de Saizieu et al., 2000; Reichmann and Hakenbeck, 2000; Miller et al., 2016). Each pheromone is typically associated with a specific receptor *blpH* allele which, in general, is restricted to respond maximally to one BlpC type (Pinchas et al., 2015). In addition, the BIR is characterized by significant diversity in bacteriocin content with 16 different putative pneumocin peptides identified to date arranged into one or two operons (Lux et al., 2007; Bogaardt et al., 2015). Many of these bacteriocins are predicted to be classified as type-IIB bacteriocins which are unmodified, two-peptide bacteriocins. Using a bioinformatics approach a recent study analyzed 4,096 distinct pneumococcal genomes categorizing genes in the BIR into a few putative functional groups based on shared bacteriocin content (Miller et al., 2016). The study concluded that the strong association between two bactericin genes could be used to predict the likelihood that they function as a pair although the predicted associations were not tested experimentally. As an example relevant to this study, there was a significant association found between the putative bacteriocin-encoding gene *blpK*, and the bacteriocin genes *blpI, blpM, blpN*, and *pncW*. Based on these associations, the authors speculated that the gene products may function as pairs in a two peptide bacteriocin.

For any given pneumococcus, the *blp* locus will encode one receptor/pheromone pair, and may contain as many as six bacteriocin genes along with their cognate immunity genes. This degree of diversity in signaling and inhibitory activity suggests that any two pneumococcal isolates might compete in any number of different ways. From a functional standpoint, only the type 6A allelic variants of the bacteriocins BlpM and BlpN have been experimentally shown to have inhibitory activity and to act together as a two-peptide bacteriocin (Dawid et al., 2007). The TIGR4 variants of BlpM and BlpN that differ from the 6A type by only 3 and 2 amino acids, respectively, have no detectable inhibitory activity in overlay assays. Whether all pneumocin peptides form two-peptide bacteriocins or whether peptides can form two peptide pairings with a variety of peptides has not been tested experimentally.

Genome data and functional analysis of strain collections have demonstrated that approximately 25% of the population encode a full length, functional BlpA transporter (BlpA+) (Miller et al., 2016). The majority of remaining BlpA non-functional (BlpA_NF_) strains have a conserved frame shift (BlpA_FS_) mutation or a large deletion in the *blpA* transporter gene (Son et al., 2011; Miller et al., 2016). These strains lack evidence of both pheromone and pneumocin secretion in agar overlay assays but retain the capacity to upregulate the locus in response to matched exogenous pheromone secreted by neighboring strains with an intact transporter gene. In addition, BlpA_NF_ bacteria are able to secrete and respond to their own BlpC under competence-inducing conditions by utilizing the alternative competence transporter, ComAB (Kjos et al., 2016; Wholey et al., 2016). The ComAB transporter can secrete pneumocins in addition to the BlpC pheromone, however, the period of secretion is limited to times of competence activation (Wang et al., 2018). BlpA+ strains have a competitive advantage during co-colonization of the mouse nasopharynx when they are competed with pneumocin sensitive strains when compared with otherwise matched BlpA_NF_ strains (Wang et al., 2018). In addition, competence induced *blp* activation is not sufficient to protect BlpA_NF_ strains from bacteriocin mediated inhibition during mouse colonization (Wang et al., 2018). It is unclear what advantage is gained by inactivation of the BlpAB transporter, however, given the prevalence of this phenotype, it has been hypothesized that BlpA_NF_ strains enjoy a fitness advantage over BlpA+ strains under non-competitive conditions.

To further understand the role of the *blp* locus in the competitive dynamics among pneumococci, we aimed to identify pneumocin genes associated with *in vitro* evidence of inhibition by screening a large pneumococcal collection for plate overlay evidence of BlpC pheromone secretion which is only seen in BlpA+ isolates under the conditions tested. Pheromone secretors were identified and analyzed for serotype, BIR content, and further screened for evidence of inhibition on agar overlay assays. Bacteriocin genes associated with inhibitory activity included the previously described *blpMN*_6*A*_ alleles and the genes encoding the putative bacteriocins BlpI, BlpJ, and BlpK. We show that BlpIJ function as a two-peptide bacteriocin that is associated with potent anti-pneumococcal inhibition. BlpK appears to function alone, however, most pneumococcal strains are immune to this bacteriocin due to BlpRH dependent and independent production of BlpK immunity. Despite the competitive advantage of BlpIJ secretion in a BlpA+ background in mouse colonization and biofilm growth and the ability of such strains to inhibit the majority of pneumococci, this combination is only rarely found in pneumococcal genomes suggesting a fitness defect within the host.

## Material and Methods

### Bacterial Strains, Growth Conditions, and Transformation

All strains and plasmids used in this study are described in Table 1, all primers listed in Table S1. Pneumococcal strains were grown in Todd-Hewitt broth supplemented with 0.5% yeast extract (THY) or on tryptic soy agar (TSA) plates supplemented with 5 μg/ml of Catalase (Worthington, Lakewood, NJ, USA) or 5% sheep blood agar plates (SBA) and incubated at 37°C in 5% CO_2_. Starter cultures were prepared for use in broth growth by cultivating in THY medium to an OD_620_ of 0.5 and then freezing in 20% glycerol. Thawed frozen starter culture were diluted 1/50 into C+Y pH 8.0 (Supplemental Material) for transformation, 1/25 into CDM media (Supplemental Material) for biofilm experiments; or 1/50 into THY for murine colonization studies. For transformation, the pneumococcal culture was allowed to reach OD_620_ = 0.15 in C+Y and further diluted 1/10 into fresh C+Y containing 1 μg/mL synthetic CSP1+2 (95% purity, Genescript, Piscataway, NJ) for 10 min at 30°C. Approximately 100 pg/ml of DNA was added to the mixture for an additional 40 min at 30°C. The culture was then incubated for 1 h at 37°C before being plated on selective media. *Escherichia coli* strains were grown in Luria-Bertani (LB) broth or LB agar supplemented with the appropriate antibiotics at 37°C. Antibiotic concentrations used were as follows: for pneumococcus 500 μg/ml kanamycin, 100 μg/ml streptomycin, 2 μg/ml chloramphenicol, 200 μg/ml spectinomycin, and 1 μg/ml erythromycin; and for *E. coli*, 50 μg/ml kanamycin, 20 μg/ml chloramphenicol, and 100 μg/ml spectinomycin.

**Table 1:** List of strains and plasmids used in this study.

**Strain #**	**Strain name**	**References**
R6x	R6 Strp^R^	Lab strain
PSD108	BlpC_6A_ reporter	Pinchas et al., 2015
PSD101	BlpC_R6_ reporter	Pinchas et al., 2015
PSD121	BlpC_164_ reporter	Pinchas et al., 2015
PMP100	BlpC_T4_ reporter	Pinchas et al., 2015
PSD120	R6 p*BIR-lacZ, blpA_*FS*_*	Wholey et al., 2016
PSD128	R6 p*BIR-lacZ, blpA::*Kan^R^-*rpsL*+	Wholey et al., 2016
P537	6A *ΔblpT-X*::Kan^R^-*rpsL*+	Son et al., 2011
P690	19A *ΔblpT-X*::Kan^R^-*rpsL*+	Son et al., 2011
P2168	19A *ΔblpT-X*::Kan^R^-*rpsL*+, *ΔblpK_*out*_/pncF_*out*_*	This study
P164	Clinical isolate	Son et al., 2011
P204	19A BIR_164_	Son et al., 2011
P279	P204 *ΔBIR*	Son et al., 2011
P912	P204 *ΔblpI::*Kan^R^*-rpsL+*	This study
P1029	P204 *ΔblpI*	This study
P572	P204 *ΔblpG_164_*	This study
P582	P204 *ΔtdpA::erm*	This study
P910	P204 *ΔblpW1/2_*P*164_*	This study
P140	Clinical isolate	Son et al., 2011
P201	19A BIR_140_	Son et al., 2011
P1213	P201 *ΔblpI*::Kan^R^-*rpsL*+	This study
P1252	P201 *ΔblpI*	This study
P1940	P201 *ΔblpI-U5*	This study
P1989	P201 *ΔblpI-J*	This study
P1245	P201 *ΔblpI-K_*in*_*	This study
P1941	P201 *ΔblpJ*	This study
P2198	P201 *ΔblpK_*in*_*::Kan^R^-*rpsL*+	This study
P2199	P201 *ΔblpK_*in*_*	This study
P2177	P201 *ΔblpI-J, K_*out*_/pncF_*out*_*	This study
A76	Clinical isolate	This study
P1214	19A BIR_A76_	This study
P1224	1214 *ΔblpK_*in*_*	This study
D39	D39 wildtype	Wholey et al., 2016
D39x	D39 Strp^R^	Wholey et al., 2016
PSD299	D39 BIR_D39_, *aad9* upstream of *blpT*, Spe^R^	Wholey et al., 2016
PSD300	D39 BIR_164_, *aad9* upstream of *blpT*, Spe^R^	Wholey et al., 2016
PSD301	PSD300 *ΔblpI*::Kan-*rpsL*+, Spe^R^	This study
PSD308	PSD300 *ΔblpA*::Kan-*rpsL*+, Spe^R^	This study
PSD309	PSD300 *blpA_*FS*_*_,,_ Spe^R^	This study
PSD310	PSD300 *ΔblpS-C*::Kan-*rpsL*+_,_ Spe^R^	This study
PSD311	PSD300 *ΔblpS-C*, Spe^R^	This study
PSD312	PSD300 *ΔblpI*, Spe^R^	This study
PSD313	D39, *iga*::Kan^R^	This study
*S. oralis*	34	Cisar et al., 1979
*S. oralis*	ATCC 35037	ATCC isolate
*S. mutans*	UA159	Ajdic et al., 2002
*S. mitis*	ATCC 49456	ATCC isolate
*S. gordonii*	ATCC 35105	ATCC isolate
*S. sobrinus*	ATCC 33478	ATCC isolate
*S. pyogenes*	HSC5 (M14 type)	Port et al., 2013
*S. pyogenes*	SF370 (M1 type)	Ferretti et al., 2001
*S. pyogenes*	JRS4 (M6 type)	Port et al., 2015
*S. pyogenes*	MEW114 (M28 type)	Jacob et al., 2016a
*S. pyogenes*	MGAS315 (M3 type)	Beres et al., 2002
*S. pyogenes*	MEW427 (M4 type)	Jacob et al., 2016b
*S. agalactiae*	ATCC 12386	ATCC isolate
*E. faecalis*	ATCC 29212	ATCC isolate
*E. faecalis (VRE)*	ATCC 51299	ATCC isolate
*Listeria monocytogenes*	10403S	Orsi et al., 2015
*Lactococcus lactis*	ATCC 14365	ATCC isolate
**Plasmids**		**References**
pEVP3	Reporter plasmid. Cm^r^	Claverys et al., 1995
pCR2.1	Cloning plasmid from TOPO cloning kit	Invitrogen
pUC19 SpeR	pUC19 derivative with *aad9* gene (spectinomycin resistance) in place of *bla* gene.	Maricic et al., 2016
pE54	pEVP3 derivative to create *blpG_*P*164_* knockout internal fragment cloned between NsiI and XbaI sites.	This study
pE55	pCR2.1 with erm cassette inserted into PCR insert of P164 *tdpA*	This study
pE82	pUC19-speR with P164 *blpA* to *blpW_2_* cloned into SmaI site	This study
pE90	pCR2.1 with *blpW_1,2_* from P164	This study
pE118	pUC19 with P164 and *blpI* deletion	This study
pE119	pCR2.1 plasmid with *blpW_1,2_,*deletion	This study
E160	pE82 with *blpI-K* deletion cloned between NsiI and EcoRI sites	This study

### Clinical Sample Collections and Serotyping

We screened a total of 454 pneumococcal isolates from three collections including: 381 clinical specimens collected between the years 2004–2006 from the Microbiology laboratory at the University of Michigan Health Systems located in Ann Arbor, Michigan, 22 pneumococcal isolates from a previous study of colonization patterns from daycare attendees in Ann Arbor, Michigan (St Sauver et al., 2000); and 54 previously described invasive and colonizing isolates from South Africa (Son et al., 2011) (IRB references in Supplemental Material). Both of the daycare and the South Africa collections were collected before PCV7 vaccine was introduced in those locations. The University of Michigan clinical isolates collection consists of samples isolated from blood, CSF, upper and lower respiratory secretions, pleural fluid, synovial fluid, bone, and other tissues and identified by standard culture techniques as *Streptococcus pneumoniae*. Pneumococci were serotyped by multiplex PCR using oligonucleotide primers for detecting 41 pneumococcal serotypes as per the US isolate scheme (da Gloria Carvalho et al., 2010), Quellung method and latex agglutination were performed for verification using specific antisera (Statens Serum Institut, Copenhagen, Denmark).

### RFLP Analysis of the BIR, *blpC* Sequencing and Detection of the *blpA* 4 bp Repeat

The DNA fragments of BIR were PCR amplified using primers 1 and 2, which bind to the highly conserved regions within *blpA* and *blpY* genes, respectively. PCR products were purified and the product was digested with restriction enzyme AseI. The size pattern of each isolate was compared to the predicted patterns from the known distinct BIR regions from the sequence database (Table S2). Isolates with identical patterns were assigned to the same group. We confirmed the absence of a *blpA* repeat or deletion in all strains with pheromone signaling as follows: a 761 bp region of *blpA* was amplified with primers 3 and 4. The product was digested with enzyme Cac8I, which will only cleave a product containing the common frame shifting 4 bp repeat (AAGC) in the *blpA* gene. The DNA fragment containing a frame shift repeat would separate into two pieces of fragments while the wild type sequence would remain intact as a single piece fragment.

Construction of deletion mutant strains can be found in the Supplemental Material.

### Overlay Assays

Overlay assays were performed as previously described (Maricic and Dawid, 2014). Briefly, strains to be assayed for inhibitory activity were spiked into TSA plates supplemented with 5 μg/ml of catalase using small sterile pipet tips. Plates were incubated for 5 h at 37°C in 5% CO_2_ to allow for growth. A volume of 300 μl of the overlay strains grown to an OD_620_ of 0.3–0.5 was added to a mixture of 100 μl of 1 mg/ml catalase, 5 ml of THY and 3 ml of molten TSA. This soft agar mixture was carefully dripped over the top of the spiked plate, which was then incubated overnight at 37°C in 5% CO_2_. For inhibitory overlay, the killing activity was indicated by a zone of clearing around the spiked site and the immunity was indicated by the growth surrounding the spiked bacteriocin producer. For BlpC pheromone secretion overlays, different BlpH type BIR*lacZ* reporters were used as overlay strains (Pinchas et al., 2015). Reporter strain PSD108 for BlpC_6A_, strain PSD101 for BlpC_R6_, strain PSD121 for BlpC_164_, and strain PMP100 for BlpC_T4_. A volume of 40 μl of X-Gal at 40 mg/ml was added to the reporter-containing soft agar before pouring. Detection of a blue zone indicates BlpC secretion is sensed by the BIR*lacZ* reporter strain. All overlays were repeated at least three separate times with strains spiked in triplicate to ensure reproducibility. Photos in figures are representative results.

### Biofilm Model

Establishment of a biofilm was performed as previous described (Marks et al., 2012). Briefly, pneumococcal D39 derivative cultures were diluted 1/25 into chemical defined media (CDM), grown to an OD_620_ of 0.5, then further diluted 1:500 into pre-warmed 34°C CDM. Pneumocin producer (PSD300 Sp^R^), pneumocin producer with the *blpA*_*F*.*S*_ mutation (PSD309 Sp^R^), pneumocin producer with Δ*blpI* strain (PSD312 Sp^R^), pneumocin producer with Δ*blpSRHC* strain (PSD311 Sp^R^), or non-producer (PSD299 Sp^R^) was co-inoculated with D39 sensitive (PSD313 Kan^R^) strains and allowed to form a biofilm on fixed H292 human lung epithelial cells grown on top of glass coverslips at 34°C + 5% CO_2_ in 24 well plates. Growth media was changed every 12 h for 3 days. For recovery of the biomass, each well was washed with PBS three times and the samples were resuspended in a final volume of 500 μl of PBS. Plates were sealed with parafilm and sonicated for 12 s in a sonication bath to disperse the biofilm. Bacterial composition of the biofilms was determined by CFU counts on single or dual selective plates. Competitive index was calculated by dividing the output ratio to the input ratio. Data from 3 independent experiments were pooled together. Kruskal-Wallis tests for multiple comparisons were performed to determine statistical significance as indicated.

### Identification of Strains Encoding BlpA and the BlpIJ Operon From Defined Strain Collections

To identify genomes from previously defined collections predicted to encode a full length BlpA, the BlpA protein sequence from strain P1031 was used in the BLAST search engine and the search was limited to the Bioprojects of the well-described Maela collection PRJEB2357, PRJEB2393, PRJEB2395, PRJEB2397, PRJEB2479, or PRJEB2480. This collection consists of the genome sequences of 3,085 colonizing isolates from infants and mothers residing in a refugee camp in Maela near the border of Myanmar and was described in Chewapreecha et al. (2014). All matches of at least 95% identity with the full-length protein were selected, this eliminated ComA sequences, as this homolog shares approximately 66% identity across the length of the protein. Duplicated sequences for an individual strain (due to repeated content in contigs) were eliminated by matching each accession number with its strain designation; any duplicated strains were counted only once. To identify the *blpI, pncBC, blpJ* region, the DNA encoding the four ORFs, 934nt from the prototypic strain TIGR4 was blasted against the above Bioprojects. Assemblies with 99–100% identity in the entire length of that fragment were used to obtain the matching strain designation. Some strains were not counted because contigs were too short to include the entire cluster. Inclusion was confirmed by protein blast against the BlpI sequence. As above, duplicated strains were counted once. Strains with matches but not appearing in Table S1 from Chewapreecha et al. (2014) were eliminated from consideration. Information of strain serotype was deduced from Table S1 of Chewapreecha et al. (2014). An identical approach was used to screen the Boston isolate collection, a collection of colonizing strains from children residing in Boston just after the release of PCV7 (Croucher et al., 2013, 2015). However, in this case, only full length BlpA and BlpI were individually searched for in the Bioproject containing all of the Boston isolate genome data, PRJEB2632. The BlpK sequence was searched for using an identical strategy in Bioproject PRJEB2632 alone. Using an identical approach, the prevalence of full length BlpA was determined in the NT collection by searching in Bioproject PRJEB2340 (Hilty et al., 2014).

## Results

### Characterization of *blp* Locus From Patient Isolates

To characterize active pneumocin production locus in the disease-causing and colonizing *Streptococcus pneumoniae* population, we screened 454 isolates from three collections: (1) 22 distinct isolates isolated from the nasopharynx of daycare attending children in the pre-PCV7 era (St Sauver et al., 2000), (2) 381 clinical isolates that were identified as pneumococcus in the microbiology laboratory at the University of Michigan Health System during a period between 2004 and 2006, (3) 51 previously described colonizing and invasive isolates from South Africa (Son et al., 2011). Using pheromone type specific reporters in overlay assays (Pinchas et al., 2015), we identified 48 strains (11%) with active BlpC pheromone secretion listed in Table 2, consistent with a *blp* locus that was functional under the *in vitro* conditions tested. The four reporter strains used in the overlays detect the four major BlpC types: 164, R6, 6A, and T4. Because BlpC_6A_ can cross stimulate BlpH_T4_ reporters, strains were designated as BlpC_6A_ secretors if they stimulated BlpH_6A_ or BlpH_T4_; strains that could only stimulate the T4 reporter were designated as BlpC_T4_ secretors (Pinchas et al., 2015). BlpC secreting strains were further characterized for capsule type, *blpA* integrity and BIR content using PCR and RFLP analysis which was used to assign bacteriocin content based on AseI digest patterns predicted for known loci.

**Table 2:** Characteristic of strains with active *blp* loci identified from clinical and colonizing isolate collections.

**Group#**	**Strain**	**Inhibition**	**BlpA**	Predicted bacteriocins^*^	***blpMN* allele**	**BlpC**	**Serotype**	**Isolation site^¥^**	**Collection^e^**
1	155	+	+	IJ, W1/2		P155	45	NP	SA
1	164	+	+	IJ, W1/2		P164	6B	CSF	SA
2	140	+	+	IJ, K		R6	35B	NP	SA
3	133	+	+	IJ, K, MNO	T4	R6	6A	NP	SA
3	158	+	+	IJ, K, MNO		6A	6A	Blood	SA
3	730	+	+	IJ, K, MNO		6A	33	NP	SA
4A	131	+	+	K, MNO		6A	23F	NP	SA
4A	132	+	+	K, MNO		6A	29	NP	SA
4B	148	+	+	K, MNO	T4	6A	14	NP	SA
4B	A31	+	+	K, MNO	T4	T4	19F	BAL	UM
4B	A76	+	+	K, MNO	T4	T4	14	Blood	UM
4B	A8	+	+	K, MNO	T4	T4	14	Blood	UM
4B	D27	+	+	K, MNO	T4	T4	7C	eye	UM
5A	135	+	+	MNO	6A	P164	6A	NP	SA
5A	776	–	+	MNO	T4	R6	19A	NP	DC
5A	C66	+	+	MNO	6A	P164	11A	Sputum	UM
5A	D57	+	+	MNO	6A	P164	11A	BAL	UM
5A	D7	+	+	MNO	6A	P164	11A	Sputum	UM
5A	E34	+	+	MNO	6A	P164	11A	BAL	UM
5B	C23	+	+	MNO	6A	T4	6A/B	Eye	UM
6	159	–	+	Q, MNO	T4	6A	23F	Blood	SA
6	171	–	+	Q, MNO	T4	6A	6A	Blood	SA
6	725	–	+	Q, MNO	T4	6A	6B	NP	DC
6	737	–	+	Q, MNO		6A	6B	NP	DC
6	739	–	+	Q, MNO	T4	6A	NT	NP	DC
6	749	–	+	Q, MNO		6A	6A	NP	DC
6	A1	–	+	Q, MNO	T4	R6	6A/B	Blood	UM
6	B61	–	+	Q, MNO	T4	6A	31	Sputum	UM
6	C69	–	+	Q, MNO	T4	6A	19A	Sinus	UM
6	C49	–	+	Q, MNO	T4	6A	6A/B	Blood	UM
6	E62	–	+	Q, MNO	T4	T4	15A	BAL	UM
7	124	–	+	Q, MNW		P164	6B	NP	SA
7	146	–	+	Q, MNW		P164	6B	NP	SA
7	173	–	+	Q, MNW		P164	6B	Blood	SA
7	723	–	+	Q, MNW		P164	6B	NP	DC
7	724	–	+	Q, MNW		P164	6B	NP	DC
7	735	–	+	Q,MNW		P164	6B	NP	DC
7	738	–	+	Q, MNW		P164	6B	NP	DC
7	A22	–	+	Q, MNW	M-6A N-T4	P164	6A/B	Blood	UM
7	B24	–	+	Q, MNW	T4	P164	6A/B	Sputum	UM
7	B60	–	+	Q, MNW		P164	6A/B	Sputum	UM
7	C61	–	+	Q, MNW		P164	6A/B	Sputum	UM
7	E45	–	+	Q, MNW	T4	R6	6A/B	Sputum	UM
8	143	–	+	ND		6A	19F	NP	SA
8	157	–	+	ND		6A	19F	Blood	SA
8	162	–	+	ND		6A	19F	Blood	SA
8	163	–	+	ND		6A	19F	Blood	SA
9	144	–	+	ND		R6	6A	NP	SA

* *Operons are separated by commas and represent Blp peptide designations. ND, not determined*.

¥ *NP, nasopharynx; BAL, bronchoalveolar lavage; CSF, cerebral spinal fluid*.

e *SA, South Africa; UM, University of Michigan; DC, Daycare*.

The 48 strains with *blp* loci that were functional for pheromone secretion in plate assays were separated into 9 groups based on RFLP predicted bacteriocin content (Table 2, Table S2). In some cases, whole or portions of the BIR were sequenced for confirmation. We tested the inhibitory activity of all stains using plate overlay assays against complete *blp* deletion mutants in two distinct genetic backgrounds: 6AΔ*blpT-X* and 19AΔ*blpT-X*. Both carry an identical allelic replacement that removes the entire *blp* locus, including all of the genes encoding regulatory and secretion proteins in addition to the entire BIR to the start codon of *blpY* (Maricic and Dawid, 2014).

Five BIR groups had evidence of inhibitory activity on plate overlay assays. For inhibitory groups 1–3, all were predicted to encode the putative pneumocins BlpI and BlpJ in the first operon. Both group 1 isolates had been previously sequenced. This group has two unique putative bacteriocins (here referred to as BlpW1_P164_ and BlpW2_P164_) following the *blpIJ* genes. The only group 2 isolate was also fully sequenced. This isolate only encodes predicted bacteriocins in the first of three BIR operons, this operon contains the *blpIJK* cluster. The Group 3 BIR has *blpIJK* followed by a second operon with *blpMNO*. Group 4 has BlpK alone in the first operon followed by BlpMNO. Group 5 had BlpMNO as its first and only operon and had a mixture of inhibitory and non-inhibitory strains. The *blpMN* alleles of group 5 were sequenced to determine if allelic differences in the bacteriocins accounted for the activity. Consistent with previous results, inhibitory activity attributable to BlpMN was only noted in strains with the *blpMN*_6*A*_ alleles.

For the non-inhibitory groups 6 and 7, both are predicted to encode BlpQ in the first operon followed by either BlpMNO or BlpMNW, respectively, in the second operon. We sequenced a selection of *blpMN* alleles in these strains and none carried the MN_6A_ alleles. The members of groups 8 and 9 were non-inhibitory and had a BIR RFLP with no match with the known sequence database and were not further analyzed.

We chose strains P164, P140, and A76 for further deletion analysis given their clear, *blp* dependent inhibitory activity on overlay plates and distinct BIR content. Group 5 isolates were not chosen for further analysis given the previously established association of the *blpMN*_6*A*_ alleles with inhibition. The BIR content of strains P164 and P140 was previously confirmed by sequencing (Son et al., 2011). BIR content for strain A76 was confirmed by sequencing in the current study and was found to be identical to the locus found in the sequenced strain Inv200 (GenBank FQ312029). The BIR regions of P140, P164, and A76 were moved into an otherwise isogenic, non-inhibitory, genetically tractable, 19A serotype background to ensure that any inhibitory activity was derived from the *blp* locus.

### Inhibitory Activity of P164 Requires BlpI but Not the Unique Accessory Proteins

**Figure 1:**
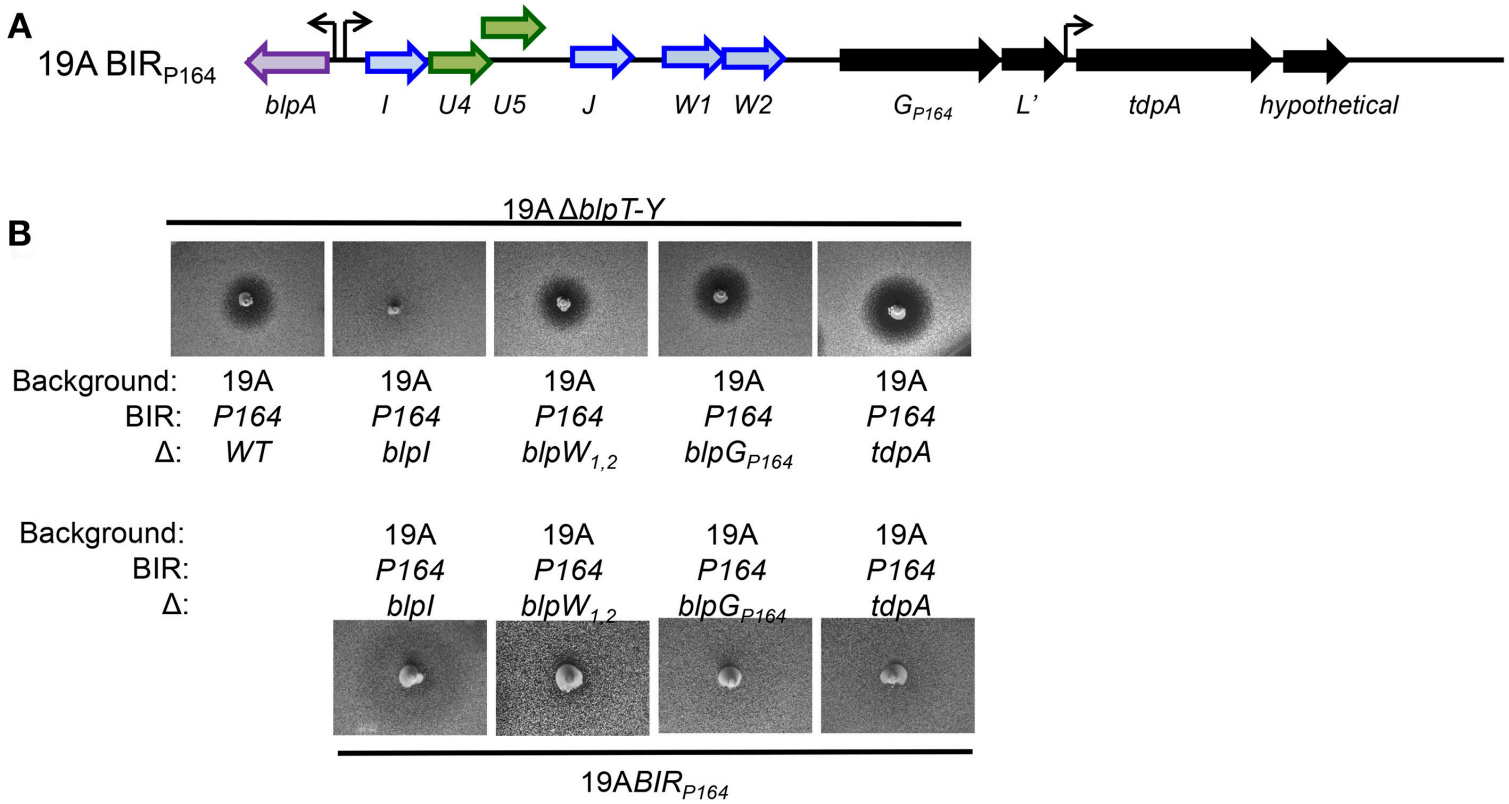
Functional analysis of the BIR region of P164 strain after moving into a 19A strain background. **(A)** Schematic representation of BIR_P164_. Blue arrows indicate predicted pneumocins, and green arrows indicate predicated immunity proteins. Black arrows indicate ORF encoding proteins of unclear significance. Curved arrows represent predicted transcriptional start sites based on the presence of a predicted BlpR binding site. **(B)** Agar overlay assays showing inhibitory and immune phenotypes. The properties of strains spiked into the plate are shown below the photo, overlay strains designations are shown above the photo.

The BIR_P164_ contains some unique genes that may influence either inhibitory activity or immunity (Son et al., 2011). The first operon in the BIR encodes the common putative bacteriocins, BlpI and BlpJ followed by two unique bacteriocin genes, *blpW1/2*_*P*164_ and a predicted protease (here called BlpG_P164_). The second operon encodes a *tdpA-*like gene predicted to encode a thioredoxin fold domain containing protein (Figure 1A). Both *blpW1/2*_*P*164_ genes are predicted to encode a peptide with a leader sequence followed by a double glycine motif typical of bacteriocins but no homology was identified in the C-terminal structural peptide. BlpG_P164_ is predicted to be an Abi type metallo-protease. Abi proteases are found within the BIR in a few sequenced genomes and at the end of the *blp* locus in nearly all strains (BlpY and PncP) (Bogaardt et al., 2015). These proteins have been proposed to confer bacteriocin immunity by processing or degrading peptides, although functional evidence is lacking (Kjos et al., 2010). The sequence of BlpG_P164_ is distinct from all other sequenced forms of BlpG. The TdpA-like protein contains an LPXTG motif, which predicts attachment to the cell wall for expression on the outer cell surface. Because the TdpA-like protein in P164 contains a thioredoxin domain and cell membrane anchor, we hypothesized that it may have a role in enhancing the function of secreted bacteriocins by forming intra or inter-molecular disulfide bonds.

To determine the role of each of the P164 BIR ORFs, we created a series of deletion mutants of P164 BIR in the 19A serotype background strain and tested these in overlay assays for inhibition and sensitivity. Pheromone secretion was confirmed in all mutants to ensure that the *blp* locus was active. Unmarked in-frame deletion mutants were created in *blpI* and *blpW1/2*_*P*164_. Insertion mutants were created in *blpG*_*P*164_ and the *tdpA* homolog. Strains were spiked into agar plates and overlaid with 6AΔ*blpT-X* and 19AΔ*blpT-X* to evaluate for inhibition while strains were inoculated into the overlay over spikes of strains with the intact locus to evaluate for immunity. The 19A-BIR_P164_ with an in-frame unmarked deletion of the *blpI* gene (Δ*blpI* strain) lost all inhibitory activity, suggesting that BlpI is at least one component of a functional pneumocin and that the BlpW1/2 peptides do not contribute to inhibition in the absence of BlpI (Figure 1B, upper). Strains lacking both *blpW1/2*_*P*164_ genes *(*Δ*blpW* strain) showed no change in inhibitory activity, suggesting that the product of these genes do not contribute to inhibitory activity (Figure 1B). Neither disruption in *blpG* or *tdpA* altered inhibitory activity or immunity (Figure 1B). These results demonstrate that the accessory proteins do not play an appreciable role in the inhibitory activity or immunity of the P164 locus.

### BlpI and BlpJ Make Up a Functional Two-Peptide Bacteriocin, and *blpU4* and *blpU5* Are the Paired Immunity Genes

**Figure 2:**
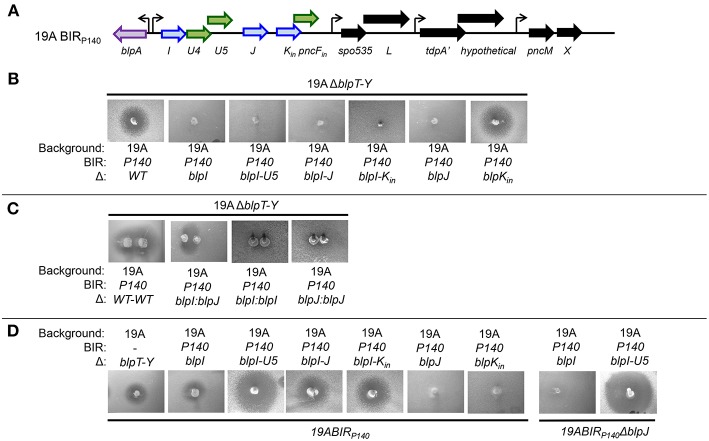
Functional analysis of the BIR region of P140 strain after moving into a 19A strain background. **(A)** Schematic representation of BIR_P140_. Blue arrows indicate predicted pneumocins and green arrows indicate predicated immunity proteins. Black arrows indicate ORF encoding proteins of unclear significance. Curved arrows represent predicted transcriptional start sites based on the presence of a predicted BlpR binding site. **(B–D)** Agar overlay assays showing inhibitory and immune phenotypes. The properties of strains spiked into the plate are shown below the photo, overlay strains designations are shown above the photo.

P140 is a colonizing isolate from the South African strain collection that was characterized by inhibition of the majority of pneumococci in the collection (Son et al., 2011). This strain has the bacteriocin cluster *blpIJK* in the first and only operon in the BIR (Figure 2A). Unmarked in-frame deletions were created in the 19A background to determine which genes in the BIR_140_ were required for inhibition and immunity. Strains with Δ*blpIJK*, Δ*blpIJ* or Δ*blpI* and Δ*blpJ* had no inhibitory activity against the 19AΔ*blpT-X* strain. The Δ*blpK* strain alone showed no defect in inhibitory activity (Figure 2B). When mutants with Δ*blpI* or Δ*blpJ* were spiked adjacent to each other on a plate, a zone of inhibition was seen in the region where the diffusion zones would overlap (Figure 2C). These results demonstrate that BlpIJ function as a two peptide bacteriocin where both peptides are required for inhibition. Because we did not perform biochemical analysis of the peptides, their active structure is not known, however, there are no modification enzymes within the locus that would predict post-translational modifications.

Producer bacteria are protected from the effects of their own bacteriocins via the production of specific immunity proteins that are typically co-transcribed with the genes encoding the bacteriocins (Jack et al., 1995). All strains with a deletion in the *blpU4*-*U5* genes found between the *blpI* and *blpJ* ORFs were inhibited by BlpIJ pneumocin producing strains (Figure 2D), indicating that one or both encode immunity peptides that protect against BlpIJ. Consistent with this, the Δ*blpJ* deletion strain can inhibit the growth of the Δ*blpI-U5* strain despite lacking inhibitory activity against any other strain including the Δ*blpT-X* strain. This finding demonstrates that the complementation between secreted BlpI (from the Δ*blpJ* spiked strain) and BlpJ (from the Δ*blpI-U5* overlay strain) occurs in the overlay and the overlay strain is inhibited because it lacks *blpU4/U5* immunity for self-protection (Figure 2D).

### BlpK Alone Is a Functional Pneumocin

**Figure 3:**
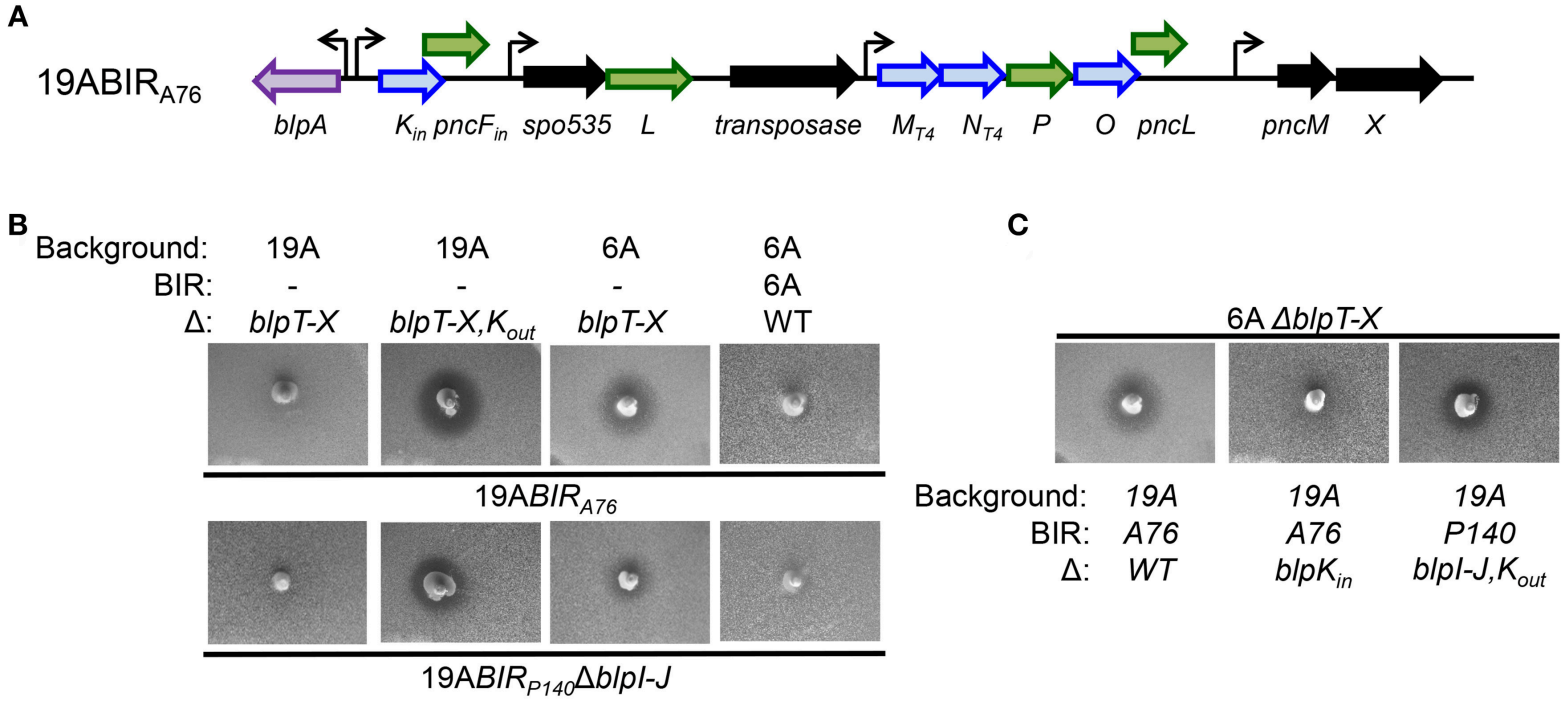
Functional analysis of the BIR region of A76 strain after moving into a 19A strain background. **(A)** Schematic representation of BIR_A76_. Blue arrows indicate predicted pneumocins, and green arrows indicate predicated immunity proteins. Black arrows indicate ORF encoding proteins of unclear significance. Curved arrows represent predicted transcriptional start sites based on the presence of a predicted BlpR binding site. **(B,C)** Overlay assays showing the inhibitory activities of BlpK-expressing stains. The properties of strains spiked into the plate are shown below the photo, overlay strains designations are shown above the photo.

A76 is a serotype 14 strain that was isolated from blood. The A76BIR has two bacteriocin operons, one encoding *blpK* and the second with *blpMNO* (Figure 3A). Overlay assays on the serotype 19A transformant carrying the A76 *blp* locus showed that this strain lacked inhibitory activity against the 19AΔ*blpT-X* deletion strain but had clear activity against the 6AΔ*blpT-X* background (Figure 3B). We observed the same pattern of inhibition using the 19ABIR_P140_ strains lacking *blpIJ* that would express only the BlpK bacteriocin. This inhibitory activity was not seen in the 19ABIR_P140_ strain with a deletion in *blpIJK*. This result suggested that BlpK bacteriocin expression in the 19ABIR_140_ Δ*blpIJ* strain and the 19ABIR_A76_ strain is responsible for inhibition of the 6A serotype Δ*blpT-X* strain. Of note, the BIR_A76_ encodes the *blpMN*_*T*4_ allele that lack inhibitory activity in all other backgrounds. In agreement with this, the deletion of the *blpK* gene in strain 19ABIR_A76_ resulted in a complete loss of inhibitory activity in plate overlay assay against the 6AΔ*blpT-X* strain (Figure 3C). These results demonstrated that BlpK is a functional pneumocin that is responsible for the inhibitory activity of strain 19ABIR_A76_ and 19ABIR_140_Δ*blpIJ* against the 6AΔ*blpT-X* strain. Given that 19ABIR_140_ Δ*blpIJ* only contains the bacteriocin *blpK* gene in the BIR, we concluded that the BlpK pneumocin peptide does not pair with another bacteriocin and functions alone in providing the observed inhibitory activity.

### *pncF_*out*_* Encodes an Immunity Protein for BlpK

Our inhibitory overlay analysis showed that the 19AΔ*blpT-X* strain is immune to BlpK expressing strains while the 6AΔ*blpT-X* strain is inhibited (Figure 3B). Because both pneumocin sensitive strains carry identical deletions of the entire *blp* locus spanning from upstream of *blpT* to just 5' of *blpY* including all regulatory, secretion and BIR genes; the immunity to the BlpK bacteriocin in the 19A background must be independent of the *blp* locus and BlpC/H signaling. Many pneumococcal genomes have two nearly identical copies of *blpK* and its co-transcribed gene *pncF* (Lux et al., 2007). One copy is often found in the first operon of the BIR as in A76 and P140 (here called *blpK*_*in*_), and the other copy is found distant from the *blp* locus (here called *blpK*_*out*_). In many studied genomes, *blpK*_*out*_*/pncF*_*out*_ is found adjacent to the genes encoding the competence transporter *comAB*. We sequenced the *blpK*_*out*_*/pncF*_*out*_ region of each deletion strain and found that the 6AΔ *blpT-X* strain that is inhibited by the BlpK expressing strains would be predicted to encode a truncated BlpK_out_ due to a premature stop codon within the coding sequence. The 19AΔ *blpT-X* deletion strain that is not inhibited by BlpK expressing strains carried an intact *blpK*_*out*_ ORF. Although all *blp* genes were originally identified by their activation by the BlpR regulator, previous work suggested that some transcription of *blpK*_*out*_*/pncF*_*out*_ might be independent of the activity of the *blp* locus (Lux et al., 2007). This group showed that transcription of the *blpK*_*out*_ gene could be appreciated in strains carrying a disruption of the BlpR regulator (Lux et al., 2007). Given the sequence identity between *pncF*_*out*_ and *pncF*_*in*_ and their presumed role encoding BlpK specific immunity proteins, we hypothesized that in the 19AΔ*blpT-X* strain, PncF_out_ can serve as an immunity protein against the inhibitory activity of the BlpK_in_ bacteriocin. The disruption in transcription/translation of the mutated *blpK*_*out*_ gene in the 6AΔ*blpT-X* strain might result in a decrease or lack of expression of *pncF*_*out*_, explaining its sensitivity to BlpK. To test this, we created a deletion of both *blpK*_*out*_ and *pncF*_*out*_ genes in the 19AΔ*blpT-X* strain (19AΔ*blpT-X,K*_*out*_) and tested this strain's immunity activity against BlpK expressing strains. Strain 19AΔ*blpT-X,K*_*out*_ was found to be susceptible to inhibition by the BlpK expressing 19ABIR_A76_ and 19ABIR_P140_ Δ*blpIJ* strains (Figure 3B). This result demonstrates that PncF_out_ can serve as the immunity protein for the BlpK_in_ pneumocin and that *pncF*_*out*_ derived immunity is effective even when the regulatory genes controlling the *blp* locus are inactive. The 6A wildtype (*blp*+) strain does not encode BlpK_in_ but showed immunity against both 19ABIR_A76_ and 19ABIR_140_ΔIJ strains (Figure 3B), suggesting that the immunity protein encoded in *pncF*_*out*_ downstream of the truncated *blpK*_*out*_ gene might only be produced in sufficient quantities to provide protection when the locus is upregulated by BlpR. It is also possible that there is another general immunity protein encoded in the *blp* locus.

To test the possibility that inhibitory BlpK_in_ activity is augmented by BlpK_out_ expression, we removed the *blpK*_*out*_/*pncF*_*out*_ genes from 19ABIR_P140_ Δ*blpIJ* strain that contained only *blpK*_*in*_ in the BIR. This mutant showed no change in inhibition against 6AΔ*blpT-X* sensitive strain compared with its parent 19ABIR_P140_Δ*blpIJ* strain (Figure 3D). This result confirmed that BlpK_in_ mediated inhibition is independent of BlpK_out_ expression. In addition, because the 19ABIR_164_Δ*blpI* strain and the 19ABIR_140_Δ*blpIJK* strains lack evidence of inhibition on plate overlay assays, there is no evidence that BlpK_out_ expression alone is sufficient for detectable *in vitro* inhibition.

### The BlpIJ Bacteriocin Inhibits the Majority of Pneumococcal Isolates

Our results so far have identified two functional pneumocins that are responsible for the inhibitory activity of three pneumococcal isolates. Because the majority of the pneumococcal population encodes the *blpK*_*out*_*/pncF*_*out*_ genes (Miller et al., 2016), BlpK pneumocin would be predicted to play less of a role in pneumococcal/pneumococcal competition. BlpIJ producing strains would be expected to inhibit any strain that lacks *blpU4/5* genes or contains these genes but fails to express them. To determine the spectrum of BlpIJ mediated inhibition, we tested the 19ABIR_P164_ strain against 368 of the clinical isolates from University of Michigan in overlay assays. Strains that did not grow well in overlays were excluded from the analysis (n = 13 isolates). The majority of pneumococcal isolates were inhibited by BlpIJ pneumocin produced by 19ABIR_P164_ strain. Only a small subpopulation of 46 isolates (12.5%) were found to be immune. We examined these isolates for the presence of the *blpU4/5* genes using restriction digestion of PCR amplified DNA region from *blpA* to start of *blpJ* gene. Forty-three immune isolates (11.7% of total) were predicted to encode *blpU4/5* genes. Only three strains had undefined immunity against the BlpIJ pneumocin.

To determine the spectrum of inhibition against other gram positive organisms, BlpIJ producing strains were tested in overlay assay against representatives of several Streptococcal species and other gram positive organisms (Table 3). D39BIR_P140_, expressing BlpIJ and BlpK was able to inhibit all tested *S. pyogenes* strains, *S. mitis, S. oralis* and *Lactococcus lactis* but not *S. gordonii, S. mutans, S. sobrinus, S. agalactiae, Enterococcus faecalis*, or *Listeria monocytogenes*. Inhibition of *S. pyogenes* and *mitis* was dependent on the expression of the BlpIJ bacteriocins as strains with individual *blpI* or *blpJ* deletions lost inhibition in overlay assays. The inhibition of *L. lactis* and *S. oralis* is dependent on a functional *blp* locus as no inhibition was noted in the 19AΔ*blpT-X* background or a background in which 19ABIR_P140_ contained a BlpA frameshift mutation. This inhibition, however, did not require any of the known *blp* bacteriocins as strain 19ABIR_P140_ΔblpI-k_in_ k_out_ lacking all known *blp* bacteriocins retained inhibition of both strains. This suggests that bacteriocins encoded outside of the *blp* locus can be secreted by the BlpAB transporter in this strain background or that the inhibition is not mediated by bacteriocins but by some other, unidentified factor produced during activation of the locus. These possibilities are currently being explored.

**Table 3:** Inhibitory profile against non-pneumococcal species.

**Serotype**	**19A**	**19A**	**19A**	**19A**	**19A**	**19A**
**BIR**	**P140**	**P140**	**P140**	**P140**	**P140**	**P140**
**Deletion**	**–**	***ΔblpI***	***ΔblpI-K***	***ΔblpT-X***	***ΔblpI-K, KoutpncF***	***ΔblpA**_***FS***_*
**SPECIES**						
*S. oralis 34*	+	+	+	–	+	–
*S. oralis ATCC 35037*	+	+	+	–	+	–
*S. mutans*	–	–	–	–	ND	ND
*S. mitis*	+	–	–	–	ND	ND
*S. gordonii*	–	–	–	–	ND	ND
*S. sobrinus*	–	–	–	–	ND	ND
*S. pyogenes M14*	+	–	–	–	ND	ND
*S. pyogenes M1*	+	–	–	–	ND	ND
*S. pyogenes M6*	+	–	–	–	ND	ND
*S. pyogenes M28*	+	–	–	–	ND	ND
*S. pyogenes M3*	+	–	–	–	ND	ND
*S. pyogenes M4*	+	–	–	–	ND	ND
*S. agalactiae*	–	–	–	–	ND	ND
*Enterococcus faecalis*	–	–	–	–	ND	ND
*E. faecalis (VRE)*	–	–	–	–	ND	ND
*Listeria monocytogenes*	–	–	–	–	ND	ND
*Lactococcus lactis*	+	+	+	–	+	–

### The BlpIJ Bacteriocin Expressed in a BlpA-Intact Background Promotes Competition

**Figure 4:**
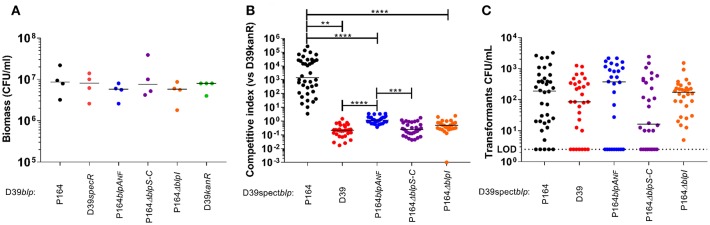
Pneumocin BlpIJ production in a BlpA+ intact strain promotes competition in dual-colonized biofilm. **(A)** Biomass of biofilm when strains were inoculated alone. **(B)** Competitive index when competitors were co-inoculated with a sensitive strain (D39kanR). **(C)** Dual Kan/Spect resistant transformants per well. LOD was 2.5 CFU/mL. There were no statistical differences between groups in A and C. Median values of 3 independent experiments are shown for each graph. Kruskal-Wallis test for multiple comparisons was used to determine statistical significance. *P*-values: ^**^0.0015, ^***^0.0004, ^****^ < 0.0001.

Pneumococcal strains are divided into BlpA-intact (BlpA+) and BlpA non-functional (BlpA_NF_) backgrounds with an approximate distribution of 25 and 75%, respectively. In plate assays, only BlpA+ strains demonstrate evidence of activation of the locus either via pheromone secretion or inhibition. Because BlpA_NF_ strains require competence induction for activation of the *blp* locus through their requirement for pheromone and bacteriocin secretion through the ComAB transporter (Kjos et al., 2016; Wholey et al., 2016), we hypothesized that plate inhibition assays might be unable to assess any bacteriocin mediated competition in these strains because the plate assays are not conducive to competence development. To determine the role of the BlpIJ pneumocin in competition in both BlpA+ and BlpA_NF_ backgrounds we performed competitive colonization studies using a previously described biofilm assay that was shown to support high levels of DNA exchange through natural spontaneous competence induction (Marks et al., 2012; Wholey et al., 2016). Because the 19A strain background did not form robust biofilms, all modified and unmodified *blp* loci were moved into the non-inhibitory D39 background, replacing the original *blp* locus. D39BIR_P164_ BlpIJ pneumocin producer strain carrying spectinomycin resistance was co-inoculated with the wildtype D39 sensitive strain carrying a kanamycin cassette (D39kan). The native D39 *blp* locus is non-inhibitory because it has both a *blpA*_*FS*_ (frame shift mutation in *blpA* gene), and a truncated BIR that lacks functional pneumocins. To ensure that any competition noted is mediated by the *blp* locus, we included as controls D39spec and versions of D39BIR_P164_ lacking either the regulatory genes (Δ*blpSRHC*) or the pneumocin gene (Δ*blpI*). Biofilms were harvested and plated on selective media after 3 days of incubation. Individually inoculated strains had similar recovered biomass from the biofilm indicating that these mutations did not alter the bacteria's ability to colonize and form a biofilm (Figure 4A). We had previously shown in plate inhibitory overlay assays that D39BIR_P164_ inhibited the growth of the parent D39 strain (Wholey et al., 2016). When inoculated into the biofilm, D39BIR_P164_ similarly out-competed D39kan (Figure 4B). Significant differences in competitive index compared with the D39spec/D39kan pairing were not observed when D39BIR_P164_Δ*blpI* or D39BIR_P164_Δ*blpSRH* were paired with D39kan. When the D39BIR_P164_*blpA*_*NF*_ strain was competed with D39kan, the competitive index was statistically higher than that noted with the D39spec or Δ*blpSRHC* strain, however, the median competitive index value of this *blpA*_*NF*_strain was significantly less than that noted with the *blpA*+ strain (Figure 4B). Transformants with dual spectinomycin and kanamycin resistance were recovered in all co-inoculated pairs confirming that the competence system was induced during biofilm formation (Figure 4C). These data demonstrate that even under competence permissive conditions, BlpIJ expressing strains with an intact BlpAB transporter have a competitive advantage over BlpA_NF_ strains that rely on the ComAB transporter for activation.

### Distribution of *blpIJ* and *blpK_**in/out**_* in Defined Pneumococcal Populations

Our results suggest that the BlpIJ bacteriocins are associated with potent anti-pneumococcal activity when expressed in strains that carry an intact *blpA* transporter gene. To determine the distribution of such strains in the overall pneumococcal population, we took advantage of and examined the genome data from the collection of 3,085 genomes that represent the pneumococcal carriage population of an isolated refugee camp in Maela and a collection of 616 strains isolated from children living in Boston for strains that had both an intact *blpA* gene and the genes encoding the BlpI and immunity proteins *blpU4/5* in the BIR (Chewapreecha et al., 2014). Out of 3,085 Maela isolates, 40% (1,240) have the genes *blpI* through *blpJ* (including *blpU4/5)* and 23% (702) are predicted to encode a full length BlpA. Only 116 isolates (4%) were predicted to encode both a full length BlpA and the genes for *blpI* through *blpJ* together (*P* < 0.0001 difference between observed and expected by chi square). Nearly identical numbers were obtained for the Boston isolates, 289/616 (47%) are predicted to encode BlpI, 152/616 (25%) are predicted to encode a full length BlpA and 27/616 (4%) are predicted to encode both (*P* < 0.0001). Consistent with our findings using the strain collection tested in this work, these data suggest that highly competitive BlpIJ producer strains are quite rare despite the competitive advantage in co-colonization.

In looking at BlpK_in/out_ distribution in the Boston collection, we found that 509/618 (82%) of genomes encoded either BlpK_in_ or BlpK_out_ or both. Sixty three percent have BlpK_out_ alone, 10% have BlpK_in_ alone and the remainder have both. The small fraction of genomes that were not found to contain any *blpK* gene might have been missed due to incomplete sequencing given the small size of the *blpK*_*out*_*/pncF*_*out*_ operon.

## Discussion

Pneumocin production by the *blp* locus is controlled by a quorum sensing regulon characterized by significant diversity in the BIR region encoding bacteriocins and immunity proteins in addition to variability in the expression of signaling pheromones and transporters. This diversity has been proposed to be the outcome of intense selective pressure induced by bacterial competition. The locus and its encoded pneumocins have been shown to provide a competitive advantage in murine models (Throup et al., 2000; Dawid et al., 2007). Through screening a large collection of isolates, here we defined the activity of two *blp* encoded pneumocins with activity *in vitro*: the two-peptide bacteriocin BlpIJ and a single-peptide bacteriocin BlpK. We had previously shown that only one of the three major allelic forms of BlpMN_6A_ is associated with inhibitory activity in overlay assays (Dawid et al., 2007). This observation was confirmed by our isolate screening; no inhibitory activity could be seen in isolates encoding other allelic forms of BlpMN. This study brings the number of *blp* encoded bacteriocins with *in vitro* evidence in inhibition to three.

BlpIJ has potent inhibitory activity against other pneumococcal isolates when expressed in a strain with an intact BlpAB+ transporter and promotes inhibition against some other Streptococcal species including *S. pyogenes* and *mitis*. Overlay assays have shown that immune pneumococcal isolates must encode the BlpU4/5 immunity proteins and have either an intact BlpAB+ system or a responsive BlpH type to that secreted by the producing strain allowing the strain to bypass the need for BlpAB mediated BlpC secretion (Son et al., 2011). Our biofilm experiments confirm that a strain with a BlpAB+ system is more competitive than an otherwise identical strain with a non-functional BlpA_FS_. The biofilm conditions allow for activation of competence and the presence of transformants confirms that the competence occurred during biofilm growth. This suggests that *com-*mediated secretion of *blp* peptides is unable to fully reproduce the *blp*-mediated competition seen in a strain that can produce both BlpAB and ComAB transporters. We recently made similar observations using a mouse model of competitive colonization (Wang et al., 2018).

Several studies of pneumococcal genomes and strain collections have demonstrated that approximately 25% of strains carry an intact *blpA* gene. Our analysis of the Maela and Boston collection of genomes demonstrated that only 4% of strains contain the *blpIU4/5J* operon together with an intact *blpA* gene. The spectrum of inhibition among pneumococci promoted by BlpIJ producing strains would be expected to be broad. Given that there are four major pherotypes that are relatively evenly distributed and that between 40 and 45% of isolates encode *blpIU4/5* (Pinchas et al., 2015; Miller et al., 2016), an additional 10% of strains would be predicted to be immune to a BlpIJ producer secreting one of the four common BlpC types. These numbers (13.8%) are consistent with the observed number of BlpIJ immune strains in the clinical isolate collection (12.5%). Only three isolates were BlpIJ immune despite lacking the linked immunity genes, *blpU4/5* by PCR screening. It is possible that these isolates do encode the immunity genes, but sequence differences resulted in the failure of PCR primers to anneal, or there may be additional mechanisms of either immunity or resistance that remain to be defined. Why *blpIJ* genes are rarely found in BlpA intact strains is not clear given a clear competitive advantage of these strains during colonization. We speculate that the large scale secretion of BlpIJ may have some inhibitory properties even on the BlpU4/5-immune producing strains, resulting in some degree of self-inhibition and a resultant fitness cost to BlpA intact strains exclusively. We have not identified experimental conditions that can demonstrate a clear fitness defect in this subset of strains to verify this hypothesis. Of note, *blpI* and *blpJ* encoding genes can be found in the genomes of closely related Streptococcal species including S. mitis (1 genome), oralis (3 genomes) and pseudopneumoniae (1 genome). These genes are associated with a region containing a highly homologous *blp* locus encoding a peptide pheromone, transporter and two-component regulatory system, consistent with either their common ancestry or horizontal gene transfer. To our knowledge, no inhibitory activity has been attributed to these loci in non-pneumococcal species although given the fact that all five have apparently intact BlpA homologs, we would expect these strains to have similar activity to the BlpIJ secreting pneumococci.

In our survey of sequenced strains we found that 71% of all BlpA+, BlpIJ predicted producing strains in the Maela collection are non-typeable (NT) although un-encapsulated isolates make up only 17% of the whole collection. Overall, 45% of NT isolates in this collection are predicted to make a full length BlpA compared with 18% of the encapsulated strains. The Boston collection contains only 14 NT isolates, but 9/14 encode a full length BlpA. In the global array of 131 NT strains analyzed by Hilty et al. (2014), 80% of sequenced isolates are predicted to encode a full length BlpA; this includes all the members of the two major NT lineages, ST344 and ST448. In the remaining “sporadic” NT isolates that were analyzed, 47% are predicted to encode a full length BlpA. It is possible that NT strains may require the competitive advantage of a fully functional *blp* locus to remain competitive in the nasopharynx in the absence of capsule. In addition, NT strains may act as DNA donors, providing genome content to virulent strains. The prevalence of the intact *blpA* gene in this background may serve as a reservoir for this allele for the entire population, explaining its persistence.

We demonstrated that BlpK secretion is associated with narrow spectrum inhibition. Bacteria producing this pneumocin are only able to inhibit pneumococcal strains with both a deletion in the *blp* locus removing all regulatory and bacteriocin genes plus a disruption in the unlinked *blpK*_*out*_/*pncF*_*out*_ gene cluster and do not promote inhibition of other tested species. No inhibition is noted in BlpK secreting strains when tested against any strain with an active *blp* locus, even those that lack *pncF*_*in*_. This finding suggests that some other component of the *blp* regulon (likely pncF_out_) protects cells against the inhibitory activity of BlpK.

The disruption in the *blpK*_*out*_*/pncF*_*out*_ operon found in our prototypic 6A strain may not be that unusual, a different amber mutation that would result in a non-functional *pncF*_*out*_ gene product was identified in 9 of 39 completed pneumococcal genomes; the remaining 30 had an intact cluster. Our data supports the findings of Lux et al. (2007) that the *blpK*_*out*_*/pncF*_*out*_ operon has *blpRH* independent expression which differentiates it from the pneumocin encoding operons in the BIR. The *blpK*_*out*_*/pncF*_*out*_ operon is preceded by a typical BlpR binding site and has been shown to be upregulated with the remainder of the *blp* locus by the addition of BlpC (de Saizieu et al., 2000), but appears to also have some degree of *blp* independent transcription. We have shown that this *blp* independent transcription is functionally relevant by demonstrating that PncF_out_ provides BlpK specific immunity even when all the regulatory *blp* genes are deleted. The *blpK*_*out*_*/pncF*_*out*_ operon is found adjacent to the *comAB* genes. We have shown that ComAB can secrete the pneumocins, but the effect of this is limited by the short duration of competence (Wang et al., 2018). It is possible that during competence induction, preformed BlpK is secreted by the newly made ComAB transporter allowing for some BlpK-mediated inhibition that cannot be seen on plates. However, given the degree of *in vitro* observed BlpK immunity in the pneumococcal population and the distribution of *blpK/pncF* genes, it seems likely that the *in vivo* target of BlpK-mediated killing is a member of the non-pneumococcal population.

We have found that strains expressing many *blp* encoded bacteriocins lack *in vitro* evidence of inhibition. In our screen the prevalent *blpQ, blpO, pncW*, and non-6A *blpMN* alleles all were not associated with an inhibitory phenotype. It is possible that the degree of inhibition promoted by these bacteriocins is too small to be observed using the plate assay. Alternatively, it is possible that we have not yet identified the target organism or the true function of the peptides.

## Conclusion

The highly adaptable pathogen, pneumococcus, competes with neighboring pneumococci and some other Streptococcal species by secreting pneumocins. We demonstrated that BlpIJ and BlpK are two functional pneumocins that demonstrate inhibitory activity when expressed in strains with intact BlpA transporter, although some competition in BlpA_NF_ backgrounds can be seen during biofilm growth. The combination of the BlpIJ pneumocin and a functional BlpA transporter gives pneumococcus a competitive advantage during colonization of a biofilm and presumably in the human nasopharynx. Despite these advantages, encapsulated strains with this combination are rare in the pneumococcal population, suggesting that the competitive advantage might be offset by energetic cost or some other effect on fitness.

## Data Availability

All genome references in the paper have already been deposited and are included in the manuscript tables.

## Author Contributions

W-YW, MA-K, and SD designed and performed the experiments and wrote the manuscript. EY, OE, and SS performed the experiments.

### Conflict of Interest Statement

The authors declare that the research was conducted in the absence of any commercial or financial relationships that could be construed as a potential conflict of interest.
