# Supplementary material for: Characterization of the Competitive Pneumocin Peptides of Streptococcus pneumoniae
Source: Front Cell Infect Microbiol. 2019 Mar 12;9:55. doi: 10.3389/fcimb.2019.00055 (PMC6422914; doi:10.3389/fcimb.2019.00055)
Supplement: Supplementary file 1 [file Table_1.pdf]

Table S1 Primers used in this study

Primer #	Use in study	Sequence
1	*blpA* F common for RFLP, confirmation *blpI* cloning, *blpKin* cloning A76	GAGGAACAAATGTACGTTTATA
2	*blpY* R common primer for RFLP	ATAGAAGTTGATACTTTTTCAT
3	in *blpA* for repeat determination	GAAGAGATTAGGGTTTTGTGCCA
4	in *blpA* for repeat determination	AGCCGCTGATGAAATGGGC
5	*blpI* deletion cloning F PstI	GGGCCCCTGCAGGTGAGCGACTTTATAGT TTCAATCCC
6	*blpI* deletion cloning R KpnR	TTCTAAAGTTCCTAAAATGATTGGTACCGG GCCC
7	*blpI* deletion inverse pcr F	CCCGGGATGCATTAAGATTATGATTCTTAA ATATA
8	*blpI* deletion inverse pcr R	CCCGGGATGCATGACAAATACTCCTTTTTT ATT
9	confirmation *blpI* cloning R	CCCGGGAGATTTCAATAATCTTGAGTAT
10	janus amplification F NsiI	GATCATGCATGTTTGATTTTTAATGGATAA T
11	janus amplification R NsiI	ACCTATGCATCTTTCCTTATGCTTTTGGAC
12	*blpW1,2* deletion cloning F	GGGCCCGGTACCCAAAAATAGGAGAATAT TTTTC
13	blpW1,2 deletion cloning R	CCCGGGATGCATGACAAATACTCCTTTTTT ATT
14	*blpW1,2* inverse PCR F smaI	GCGCGCCCCGGGGACAAATACTCCTTTTT AAAAA
15	*blpW1,2* inverse PCR R smaI	GCGCGCCCCGGGTAAAAAATCATCTAGAC ATTAAA
16	janus amplification F SmaI	GACTTGCCCGGGTCATTAGCTTTTTTAGTG GA
17	janus amplification R SmaI	GACTTCCCCGGGGAGCACTTTGTAAGTCT GTTG
18	*blpKin* clone R A76 blpL R	TCTCGCAAGGAAGATGTTCCG
19	*blpG* truncation cloning NsiI	GGGCCCATGCATGAAGAACATAAGTTTTA TCTTT
20	*blpG* turncation cloning XbaI	GGCCTCTAGACAGCTGAAAGAAAGACAGC A
21	*blpG* truncation check	GGACAATGCTAAAAAATCAT

22	*lacZ* R to confirm chromosomal integration, reverse primer to amplify *blpA::KanR-rpsL+* and $blpA_{FS}$	TCAAAAATAATTCGCGTCTG
23	*tdpA* clone F	TAAAAAGTAGAGCTTAATGAA
24	*tdpA* clone R	AAATTTTCCATCCAAATCATAA
25	outside *tdpA* clone F	TATCATGCTTCTGCTTATGT
26	outside *tdpA* clone R	CCTTAACAATGTTAGTATAC
27	in *blpA* F for blpI deletion	TTATCTTTATGGGGTTGATAG
28	in *pncC* R for *blpI* deletion	CCCGGGAGATTTCAATAATCTTGAGTAT
29	in *blpG* R for *blpI* deletion	GGCTTTTCCAAGGACGGTAG
30	*blpI-J* ko gibson upstream	TTATCTTTATGGGGTTGATAG
31	*blpI-J* ko gibson upstream	ataagtcttaTTGTTCCATCATTTTTGTATTC
32	*blpI-J* ko gibson downstream	atggaacaaTAAGACTTATAGTAAAATTATGAGGATTC
33	*blpI-J* ko gibson downstream	CTACGGAAAAATTTAAAAACTG
34	*blpJ* ko only gibson	ataagtcttaCTGTGACAACATTTTTGTATTC
35	*blpJ* ko only gibson	ttgtcacagTAAGACTTATAGTAAAATTATGAGGATTC
36	*blpKin* ko gibson upstream	CTGTTATGGATAATGAAGAACTC
37	*blpKin* ko gibson upstream janus	taaggaaagTTGTGACATCATTTTTGTATCC
38	*blpKin* ko janus gibson	atgtcacaaCTTTCCTTATGCTTTTGGAC
39	*blpKin* ko janus gibson	tatcctttttGTTTGATTTTTAATGGATAATGTG
40	*blpKin* ko gibson downstream janus	ttaaaaatcaaacAAAAAGGATACAGATAGTACCCC
41	*blpKin* ko gibson downstream	GAATACTCCTTTACATTTGTTATCTTTATC
42	*blpKin* ko gibson in frame	tatcctttttTTGTGACATCATTTTTGTATCC
43	*blpKin* ko gibson in frame	atgtcacaaAAAAAGGATACAGATAGTACCCC
44	*blpKout* ko gibson upstream	GAAACTTATGAATTACTGGCG
45	*blpKout* ko gibson upstream	attcagacaCTTTCCTTATGCTTTTGGAC
46	*blpKout* ko spect gibson	gaatcataacGTTTGATTTTTAATGGATAATGTG

47	*blpKout* ko spect gibson	aaatccataaCAATTTTTTTATAATTTTTTTAATCTG
48	*blpKout* ko gibson downstream	taaaaaaattgTTATGGATTTTAAAAGTTTTATTATTG
49	*blpKout* ko gibson downstream	CACTAATATAGGGATAAAAGAGAGC
50	*blpB* F primer to amplify *blpA::KanR-rpsL+* and $blpA_{FS}$	GATAAAAATGGCTCCTCTGC
51	*blpS-C* ko gibson upstream	CAACTCTTTCCGCTGGAC
52	*blpS-C* ko gibson upstream janus	taaggaaagTGTCTGAATAATCATATGTTTCATTG
53	*blpS-C* ko janus gibson	attcagacaCTTTCCTTATGCTTTTGGAC
54	*blpS-C* ko janus gibson	gaatcataacGTTTGATTTTTAATGGATAATGTG
55	*blpS-C* ko gibson downstream janus	aaaatcaaacGTTATGATTCTCCTTTTTGATAAG
56	*blpS-C* ko gibson downstream	CATAATACCTTTATGAATTTTACTAAAC
57	*blpS-C* ko gibson in frame	gaatcataacTGTCTGAATAATCATATGTTTCATTG
58	*blpS-C* ko gibson in frame	attcagacaGTTATGATTCTCCTTTTTGATAAG
59	*iga::kan* gibson upstream	TACCGTGTAAATTTCATGGTAG
60	*iga::kan* gibson upstream	aatcaaacggaCGCCTTTATCACTTACTACAGC
61	*iga::kan* gibson kan	tgataaaggcgTCCGTTTGATTTTTAATGGATAATG
62	*iga::kan* gibson kan	tattgccaactCTAAAACAATTCATCCAGTAAAATATAATATTTTATTTTC
63	*iga::kan* gibson downstream	aattgttttagAGTTGGCAATAAGGTAGTACAC
64	*iga::kan* gibson downstream	CTATTTTTTATTCTCAAAAATTGAACTTC
