# Supplementary material for: Characterization of the Competitive Pneumocin Peptides of Streptococcus pneumoniae
Source: Front Cell Infect Microbiol. 2019 Mar 12;9:55. doi: 10.3389/fcimb.2019.00055 (PMC6422914; doi:10.3389/fcimb.2019.00055)
Supplement: Supplementary file 2 [file Table_2.pdf]

Table S2 – Accession numbers and strain designations of database matches based on RFLP.

Group	AseI BIR fragments	GenBank acession # for genome match	Database match
1	2.3,2.1,0.5	HQ668085	P164
2	4.5,0.55	HQ668083	P140
3	3.8,1.7,0.55	AE005672	TIGR4
4A	3.2,1.7	CP018136	SP49
4B	2.5,1.7,0.7	CP001033	CGSP14
5A	2.3,0.9	CP000920	P1031
5B	3.3,0.9	ABAA01000015	SP6BS73
6	2.3,1.7,1.3	NZ_ABFS01000001	CDC1873
7	2.3,1.2,0.37	CP002176	670-6B
8	6.0,2.7	NA	NA
9	1.9,1.2	NA	NA
