# Supplementary material for: Characterization of the Competitive Pneumocin Peptides of Streptococcus pneumoniae
Source: Front Cell Infect Microbiol. 2019 Mar 12;9:55. doi: 10.3389/fcimb.2019.00055 (PMC6422914; doi:10.3389/fcimb.2019.00055)
Supplement: Supplementary file 3 [file Data_Sheet_1.pdf]

Supplemental Methods:

Contents:

1. Media Composition. Pages 1-3
2. Construction of *blp* mutants. Pages 3-8
3. IRB information for clinical isolates. Pages 8-9
4. References for supplemental section. Page 9

Media Composition-

Chemically Defined Media (CDM). Adapted from [1].

Gibco Custom Media containing:

Media Component	Concentration (g/L)
Adenine	0.02
L-alanine	0.1
L-arginine	0.1
L-aspartic acid	0.1
D-biotin	0.0002
Calcium chloride, anhydrous	0.0051
Calcium D-pantothenate	0.002
Cyanocobalamin	0.0001
L-cystine disodium salt	0.0629
Ferric nitrate, nonahydrate	0.001
Ferrous sulfate, heptahydrate	0.005
Folic acid	0.0008
L-glutamic acid	0.1
L-glutamine	0.2
Glycine	0.1
Guanine hydrochloride, anhydrous	0.0182
L-histidine	0.1
trans-4-hydroxy-L-proline	0.1
L-isoleucine	0.1
L-leucine	0.1
L-lysine	0.1
Magnesium sulfate, anhydrous	0.3419
Manganese(II) sulfate, monohydrate	0.005596
L-methionine	0.1
Niacinamide	0.001
Beta-NAD hydrate	0.0025
4-aminobenzoic acid	0.0002
L-phenylalanine	0.1
Potassium phosphate, dibasic, anhydrous	0.2
Potassium phosphate, monobasic, anhydrous	1
L-proline	0.1
Pyridoxal hydrochloride	0.001
Pyridoxine hydrochloride	0.000853
Riboflavin	0.002

L-serine	0.1
Sodium acetate, anhydrous	2.7126
Sodium phosphate, dibasic, anhydrous	7.35
Sodium phosphate, monobasic, monohydrate	3.195
Thiamine hydrochloride	0.001
L-threonine	0.2
L-tryptophan	0.1
L-tyrosine disodium salt	0.144
Uracil	0.02
L-valine	0.1
**Total**	**17.092949**

Preparation from powdered formulation to make 100mL:

1. Dissolve the following in Milli-Q water per 100 mL of final media volume:

   - 1.709 g CDM powder (Gibco Custom Media)
   - 0.25 g sodium bicarbonate
   - 0.075 g L-cysteine hydrochloride (or 0.0836 g of the monohydrate form)
   - 1 g dextrose

2. Add an appropriate volume of 10% choline chloride stock (e.g. 1 mL per 100 mL final media volume for a final concentration of 0.1% choline chloride).
3. Final pH should be 7.00-7.05.
4. Filter-sterilize and use immediately.

C+Y Formulation:

**Media component**	**Concentration**
Bacto Casamino Acids	4.32 g/L
L-asparagine	43.2 mg/L
L-cysteine hydrochloride	43.2 mg/L
L-glutamine	21.6 mg/L
L-tryptophan	4.32 mg/L

$CaCl_2$	2.3 mg/L
$MgCl_2 \times 6H_2O$	0.4 g/L
$MnSO_4 \times 4H_2O$	13.4 mg/L
$CuSO_4$	432 mg/L
$FeSO_4 \times 7H_2O$	432 mg/L
$ZnSO_4 \times 7H_2O$	432 mg/L
glucose	1.6 g/L
sucrose	0.2 g/L
adenosine	15.8 mg/L
uridine	15.8 mg/L
sodium acetate	1 g/L
sodium pyruvate	216 mg/L
biotin	0.5 mg/L
nicotinic acid	0.5 mg/L
pyridoxine hydrochloride	0.6 mg/L
D-calcium pantothenate	2.1 mg/L
thiamine hydrochloride	0.55 mg/L
riboflavin	0.24 mg/L
choline chloride	4.3 mg/L
yeast extract	0.11% (w/v)
potassium phosphate buffer, pH 8.0	50 mM

<u>Construction of *blp* mutants:</u>

Construction of deletion mutant strains. To transfer the *blp* loci of P164, P140 and A76 into 19A serotype background, cell lysates containing genomic DNA of the indicated strains were transformed into a 19A Δ*blp* strain (P690), that contains an exchangeable janus cassette (Kan^R $Strp^S$) replaced the native *blp* locus [2]. The resulting transformants 19A-BIR_{164} (P204), 19A-BIR_{140} (P201) were previously described [2]. A new strain, 19A-BIR_{A76} (P1214) was selected on streptomycin containing plates and screened for loss of kanamycin resistance indicating replacement of the janus cassette. To ensure the

entire functional *blp* locus was transformed, overlay assays were performed to examine inhibitory activity and BlpC pheromone secretion.

To construct the *blpI* deletion in 19A-BIR_{164} (P204), a region from *blpA* to *blpJ* genes was first amplified by PCR primers 5 and 6 (primer sequences listed in **Supplemental Table 1**) which created PstI and KpnI restriction sites. This fragment was then blunt end ligated into SmaI restriction site of a modified *E. coli* plasmid pUC19 vector containing a spectinomycin cassette replacement of ampicillin resistance cassette (pE82). Inverse PCR using primers 7 and 8 was performed on pE82 and the product was re-ligated to form a clean *blpI* deletion. The resulting plasmid (pE118) was confirmed by PCR with primers 1 and 9. A janus cassette was amplified using primers 10 and 11 and ligated into the NsiI restriction site of pE118. Strain P204 was first transformed with plasmid pE118-janus and selected for kanamycin resistance and streptomycin sensitivity resulting in strain P912. P912 was then transformed with pE118 and selected on streptomycin plates to replace the janus cassette with the in frame *blpI* gene deletion resulting in strain P1029. Allelic exchange (rather than plasmid insertion) was confirmed by PCR and the presence of the expected sequence was confirmed by sequencing.

To construct *blpW1/2* deletion in P204, a region around *blpW1/2* genes was amplified from P164 using primers 12 and 13 and cloned into the pCR2.1 plasmid (Invitrogen). The *blpW1/2* genes were deleted by inverse PCR using primers 14 and 15 which introduced a SmaI restriction site. The inverse PCR product was either self-ligated as plasmid pE119, or ligated with a janus cassette amplified with primers 16 and 17 and directly transformed into P204 selecting on kanamycin. Strain P204-janus was then transformed with pE119 to replace the janus cassette introducing the in-frame *blpW1/2* genes deletion. This strain was named P910, and was confirmed by sequencing.

To construct *blpG*$_{164}$ truncation in P204, the N-terminal 500 bp fragment of the *blpG*$_{164}$ gene was amplified using primers 19 and 20, digested with NsiI and XbaI restriction enzymes, and then ligated into the plasmid of pEVP3. The resulting plasmid was verified with restriction digestion and named as pE54. P204 was then transformed with plasmid pE54 and transformants were selected on chloramphenicol. The strain with integration of the plasmid creating a *blpG*$_{164}$ truncation, was named P572, and was verified using primers 21 and 22.

To construct *tdpA* deletion in P204, the DNA region around the *tdpA* gene portion of BIR$_{164}$ was amplified using primers 23 and 24, and cloned into the pCR2.1 plasmid (Invitrogen). An erythromycin (Erm) resistance cassette was cloned in between two unique internal MfeI sites, disrupting the *tdpA* gene resulting in plasmid pE55. P204 was transformed with plasmid pE55 and selected for erythromycin resistance. The correct mutant, named P582, was further confirmed for allelic replacement via PCR using paired primers 23 and 26; 25 and 26; and 23 and 25.

To delete *blpI* in 19A-BIR$_{140}$ (P201), the Δ*blpI*::janus from P912 was amplified using the primers 27 and 28 and transformed into P201 selecting on kanamycin. The resulting transformant, P1213, was confirmed by PCR. The *blpI* deletion DNA fragment from P1029 was amplified using primers 27 and 29 and transformed into P1213. Transformants were selected on streptomycin and an isolated *blpI* deletion confirmed by sequencing. The *blpI-K* deletion was constructed by transforming P1213 with plasmid pE160 that contains a *blpI-K* deletion. Plasmid pE160 was created by digesting the *blpI* deletion plasmid pE118 with NsiI/EcoRI to remove the cloned region downstream of the *blpI* start codon and ligating a

PCR amplified fragment starting downstream of the *blpK* gene. Creation of the correct deletion was confirmed by PCR and sequencing.

The in-frame deletion of *blpI-U5* (P1940), *blpI-J* (P1989), *blpJ* (P1941) and *blpK$_{in}$* (P2199) in P201 background were constructed using Gibson Assembly method [3]. The in-frame deletion was created by two-piece assembly using just the 1 kb flanking regions. Primers were PCR primers with overlapping sequences between the adjacent DNA fragments designed using NEBuilder™ webtools (New England Biolabs, USA). For deletion of *blpI-J*, primers 30 and 31 were used for upstream fragment, and primers 32 and 33 were used for downstream fragment amplifications. PCR products were purified and assembled using Gibson Assembly MasterMix (New England Biolabs, USA) according manufacture's instruction. The reaction mix was then transformed into P1213, a Δ*blpI*::janus strain. The transformant, named P1989, was selected on streptomycin and screened for loss of kanamycin resistance, indicating loss of the janus cassette. Due to promoter and peptide leader sequences similarity between *blpJ* and *blpK*, primer 32 annealed at *blpJ* non-specifically, and created *blpI-U5* deletion, named P1940 that was confirmed by sequencing. For deletion of *blpJ*, primers 30 and 34 were used for upstream fragment, and primers 35 and 33 were used for downstream fragment amplifications. Following Gibson Assembly of these two fragments. Reaction mix was transformed into P1213. The transformant, named P1941, was selected on streptomycin and screened for loss of kanamycin resistance, presence of the *blpJ* deletion was confirmed by PCR. All mutants were confirmed by sequencing. For *blpK$_{in}$* deletion, a three-pieces Gibson assembly was first constructed using upstream of *blpK* fragment amplified with primers 36 and 37, janus cassettes amplified with primer 38 and 39, and downstream of *blpK$_{in}$* fragment amplified with primers 40 and 41 and then transformed into P201. The transformant, named P2198 was selected on kanamycin containing plates indicating a janus replacement of *blpK$_{in}$*. Then, the in-frame deletion of *blpK$_{in}$* DNA fragment was assembled using upstream of *blpK$_{in}$* fragment amplified with primer 36 and 42

and downstream of *blpK*$_{in}$ fragment amplified with primers 43 and 41. The reaction mix was then transformed in to 2198 and selected on streptomycin containing plates for janus exchange, resulting transformant was named P1905. Deletions were confirmed with sequencing.

To delete *BlpK*$_{in}$ in 19A-BIR$_{A76}$ (P1214), the *blpK*$_{in}$ gene was amplified using primers 1 and 18 and cloned to pcR 2.1 TOPO Vector and transformed in *E. coli*. A spectinomycin resistance cassette was inserted into the MfeI site, a unique restriction site 75bp from the start codon of the *blpK*$_{in}$ gene resulting in a *blpK*$_{in}$ disruption. This plasmid was then transformed into P1214 and selected for spectinomycin resistance. The mutation of *blpK*$_{in}$ transformant, named P1224, was confirmed using PCR with primers 1 and 18.

For deletion of *blpK*$_{out}$ in P690 and in P1989, the Gibson Assembly method was used. A spectinomycin cassette was inserted and replaced the *blpK*$_{out}$ and *pncF*$_{out}$ genes. Primers 44 and 45 were used for upstream fragment, primers 46 and 47 were used for the spectinomycin cassette, and primers 48 and 49 were used for downstream fragment amplification. The reaction mix was transformed into P690, and P1989. Transformants, named P2168 and P2177, respectively, were selected on spectinomycin and confirmed by PCR.

For deletion of *blpI* and *blpA*$_{FS}$ mutations (PSD309) in D39 BIR$_{164}$ (PSD300) background, janus exchange was used. PSD301 containing a Δ*blpI*::janus was transformed with a Δ*blpI* deletion DNA fragment amplified from P1029 using primers 27 and 29. The resulting D39 BIR$_{164}$ Δ*blpI* strain (PSD312) was verified with PCR. The *blpA*::janus was amplified from PSD128 using primers 50 and 22; and then transformed into PSD300. The resulting janus containing strain (PSD308) was then transformed with

blpA$_{FS}$ mutation amplified from PSD120 using primers 50 and 22. The resulting D39 BIR$_{164}$ *blpA*$_{FS}$ strain (PSD309) was verified by sequencing.

For construction of Δ*blpSRHC* deletion in D39 BIR$_{164}$ (PSD300) background, and *iga::kan* mutation in D39 sensitive strains, Gibson Assembly method was used. A janus cassette replacing *blpSRHC* fragment was first assembled by amplifying an upstream fragment with primers 51 and 52, the janus cassette with primers 53 and 54, and a downstream fragment with primers 55 and 56. This reaction mix was transformed into PSD300 to obtain a *blpSRHC::janus* strain (PSD310). Then, a complete deletion of the *blpSRHC* fragment was assembled by joining the up upstream fragment amplified with primers 51 and 57; and the downstream fragment amplified with primers 58 and 56. This reaction mix was transformed into PSD310 to obtain Δ*blpSRHC* strain (PSD311). For *iga::kan* mutation, a kanamycin cassettes was inserted in the middle of *iga* gene. Primers 59 and 60 were used for upstream fragment, primers 61 and 62 were used for kanamycin cassette, and primers 63 and 64 were used for downstream fragment amplification. The reaction mix was transformed into D39. The transformant (PSD313) was selected on kanamycin.

IRB information for clinical isolate collections:

The collection of colonizing and invasive disease isolates was approved by the Human Research Ethics Committee, University of the Witwatersrand, South Africa. Written informed consent was obtained from colonization study participants or their legal guardians. Invasive isolates were acquired as part of routine clinical care and no patient information was recorded. Ethics reference number 050705. The collection of clinical isolates from the University of Michigan was acquired as part of routine clinical care and was covered under IRB HUM00035830 at the University of Michigan. IRB approval status for the daycare collection was discussed in [4]. No identifying information was accessed or utilized in this work.

[1] L.R. Marks, R.M. Reddinger, and A.P. Hakansson, High levels of genetic recombination during nasopharyngeal carriage and biofilm formation in Streptococcus pneumoniae. MBio 3 (2012).
[2] M.R. Son, M. Shchepetov, P.V. Adrian, S.A. Madhi, L. de Gouveia, A. von Gottberg, K.P. Klugman, J.N. Weiser, and S. Dawid, Conserved mutations in the pneumococcal bacteriocin transporter gene, *blpA*, result in a complex population consisting of producers and cheaters. MBio 2 (2011).
[3] D.G. Gibson, L. Young, R.Y. Chuang, J.C. Venter, C.A. Hutchison, 3rd, and H.O. Smith, Enzymatic assembly of DNA molecules up to several hundred kilobases. Nat Methods 6 (2009) 343-5.
[4] J. St Sauver, C.F. Marrs, B. Foxman, P. Somsel, R. Madera, and J.R. Gilsdorf, Risk factors for otitis media and carriage of multiple strains of Haemophilus influenzae and Streptococcus pneumoniae. Emerg Infect Dis 6 (2000) 622-30.
